# Lived Experiences of Caretakers of Dementia Patients

**DOI:** 10.1002/alz70860_103401

**Published:** 2025-12-23

**Authors:** Sudarshan Paudel

**Affiliations:** ^1^ Patan Academy of Health Sciences, Kathmandu, Nepal, Bagmati, Nepal

## Abstract

**Background:**

Dementia is becoming more common, particularly in low‐resource nations like Nepal where the burden is increased by aging populations and insufficient medical facilities. Caregivers—mostly family members—face extreme financial, emotional, and physical hardship due to a lack of support systems. Services rely on personal resilience, community networks, and traditions. This project intends to fill the information gap on dementia caregivers' experiences in Nepal by investigating their daily realities, coping strategies, and potential improvements to support networks.

**Method:**

This qualitative exploratory study explored the experiences of dementia caregivers in Nepal using purposive and snowball sampling methods. Participants were selected to ensure diversity in demographic and geographic backgrounds. Semi‐structured interviews were conducted in private settings with thirty caregivers who had at least three months of caregiving experience. Transcription and translation of the interviews were completed on the same day. Verbal consent was obtained from all participants, and ethical approval was granted by the Nepal Health Research Council (*ERB#462‐2024*). Data were analyzed using a six‐step thematic analysis approach.

**Result:**

Seven themes emerged from the analysis: Physical challenges, Psychological challenges, Social challenges, Financial burden, Learning process of the management, Facilitators for adequate care, Barriers for the adequate care and Recommendation to the policy level.

The study features the demanding role of caregivers, who are responsible for managing patients' mobility, and quality of life, while also bearing significant emotional strain. Families often view caregiving as a duty, even amidst financial hardship. Some caregivers have adjusted their living spaces to better cater to their dependents' needs. However, the lack of respite care and social support impairs these challenges. The study suggests that initiatives such as community support groups, caregiver education programs, and home‐based healthcare services should be developed to support caregivers.

**Conclusion:**

Dementia is a growing issue in Nepal, with home caregivers essential yet challenged. Strengthening support through education, resources, and formal support systems for caregivers is crucial for improving care quality. The study identifies areas for improvement and offers recommendations to enhance dementia care services.

